# Comparative Study of Number and Distribution of IgG^+^ Cells in Oral Lichen Planus and Oral Lichenoid Lesions

**Published:** 2009

**Authors:** Parichehr Ghalayani, Seyed Mohammed Razavi, Darab Gholami

**Affiliations:** * Associate Professor, Department of Oral Medicine and Torabinejad Dental Research Center, School of Dentistry, Isfahan University of Medical Sciences, Isfahan, Iran; ** Associate Professor, Department of Oral and Maxillofacial Pathology and Torabinejad Dental Research Center, School of Dentistry, Isfahan University of Medical Sciences, Isfahan, Iran; *** Post Graduate Student, Department of Orthodontics, School of Dentistry, Tehran University of Medical Sciences, Tehran, Iran

**Keywords:** Immunoglobulin G, immunohistochemistry, lichenoid lesions, oral lichen planus

## Abstract

**Background:**

Oral lichen planus is a common mucocutaneous disorder with unknown etiology. While current data suggest that oral lichen planus is a cell-mediated disease, differential diagnosis of this disease and oral lichenoid lesions is very problematic, both clinically and histopathologically. This study aimed to compare immunohistochemical features of these similar diseases.

**Methods:**

This was a descriptive-analytic study in which formalin-fixed and paraffin-embedded tissue sections of 30 oral lichen planus and 30 oral lichenoid lesions were immunohistochemically analyzed for number and distribution of IgG^+^ cells. A standard biotin-streptavidin procedure after antigen retrieval was used. Data were analyzed in SPSS software using Mann-Whitney U test.

**Results:**

There were some significant differences in distribution of IgG ^+^cells among different locations in oral lichen planus and also in oral lichenoid lesions separately; but the differences between distribution of IgG^+^ cells between the two groups of oral lichen planus and oral lichenoid lesions were not significant.

**Conclusion:**

There was no significant difference in number and distribution of IgG^+^ cells between the two groups. So, this study can suggest that location of IgG is similar in samples of oral lichen planus and oral lichenoid lesions and consequently, this marker cannot help us differentiate them from each other. Other markers can be analyzed in further studies in order to find an appropriate distinguisher between the two lesions.

## Introduction

Lichen planus is a common mucocutaneous lesion and includes about 9 percent of oral lesions. Although the etiology of this disease is unknown, degeneration of basal cell epithelium with cell-mediated immunity is a probable cause. Oral lichen planus (OLP) has clinically different figures but essentially includes three forms: keratotic, erosive and bullous. The keratotic form is the most common form; however in a study, erosive form was reported as the most common form.^1^ Microscopic view of lichen planus is not specific because cases such as lichenoid lesions induced by drugs or amalgam,^2^,^3^ lupus erythematosus and chronic ulcerative stomatitis may have similar views.^4^ Oral lichenoid lesions (OLL) are also induced by drug irritations, hepatitis C virus, allergic reactions (amalgam mercury) and graft versus host disease (GVHD).^5^ This disease occurs frequently in the 5th decade of life and is more common in females. Although these lesions may occur in every region of oral mucosa, buccal mucosa is the most common site. These lesions may accompany pain and discomfort and cause interference with work and life quality. Some theories suggest premalignancy characteristics in lichen planus lesions especially erosive form,^2^ but a recent study indicated that the likelihood of occurrence of oral cancer in patients with OLL is more than that in OLP.^6^ Meanwhile, differentiation of OLP and OLL is very difficult clinically and histopathologically.^7^,^9^ So, for differentiation of these two, the use of immunofluorescence method is recommended.^10^ In 1977, Shousha et al examined the distribution of IgG and IgM in 20 samples of OLP lesions and 5 samples of non-specific inflammations or OLL using the immuno-histochemical technique, PAP. The samples were in paraffin sections. They found that in lichen planus, immunoglobulins precipitated within and around epithelial cells, colloidal bodies, interjunction of epithelium-connective tissue and in some inflammatory cells. IgM precipitation was positive for all samples and 8 of 13 examined cases were positive for IgG^+^ cells. The peripheral epidermal cells were often negative.^7^

Bouloc et al in 1998 evaluated lichen planus and found linear IgG and C_3_ precipitation in basal membrane region in samples labeled with immunofluorescence method around dermal bolls.^11^ Seishima et al used direct immunofluorescence technique in skin around lichen planus and found linear IgG precipitation in basal membrane.^12^ The main purpose of this study was evaluation of applicant potentials of immunohistochemical method differentiating OLP from OLL. Number and distribution of IgG^+^ cells were regarded as a base of comparison. Biocina-Lukenda et al in their study evaluated IgA, IgM and IgG in the serum of patients with OLL and found significant increase in serum level of IgA and IgM in patients, but the increase in serum levels of IgG was not significant.^13^

## Materials and Methods

This was a descriptive-analytic study. The sample included 30 cases of OLP and 30 cases of OLL referred to Oral Diseases Department of Dental Faculty of Isfahan University of Medical Sciences, from 1987 to 2005. Biopsies from all patients’ lesions were prepared and samples were approved histopathologically by an oral pathologist. After evaluating the patients’ files, the lesions were differentiated into two groups (each included 30 cases) of OLP and OLL. The inclusion criteria included bilateral lesions, reticular form or combination of other forms of lichen planus with reticular form, lack of history of diabetes, high blood pressure, oral medications specially non steroidal anti inflammatory drugs (NSAIDs), hepatitis B and C (which were confirmed with the required laboratory tests), grafts, likelihood of GVHD, amalgam fillings and history of dermal popular lesions accompanying oral lesions. If all of these factors existed, the lesion was classified as OLP and if lesions reported unilaterally or specially as erosive and bullous or if one or more factors mentioned accompanied by oral lesions, the lesion was classified as OLL. Fixed paraffinic blocks of OLP and OLL were prepared from all patients in Pathology Department of Dental Faculty of Isfahan University of Medical Sciences. Samples without required quality and also samples diagnosed improper by pathologist in histological (H & E) or immunohistochemical studies were excluded from the study. Given all the above information, samples were classified into two groups of OLP and OLL. Some of the criteria of a proper sample included: sufficient epithelium, intact connective tissue, lack of its rupture from epithelium and lack of tissue wrinkling, etc. After impression, samples were sectioned into 3-4 micron thicknesses by microtome (Erma, Japan). Then, the sections mounted on slides by Poly-L-Lysine (for better maintenance and prevention of tissue rupture). The mounted slides were taken into room temperature for 12-24 hours (air drying); then, maintained in room temperature or 2-8°C until the time of staining. In this stage, the slides were kept at 60° C for 45 minutes and then, 3 minutes in xylol changes for removing paraffin and 5 minutes in alcohol changes (descending) until distilled water for rehydration were done. Then, samples were taken into citrate buffer with pH = 6 for fixing antigens and these complexes were put into microwave (W = 750) to nor-malize molecular structure of antigen, which was distorted as a result of fixation, with controlled temperature. In the next stage, samples were cooled in room temperature for 20 minutes and after immunohistochemical staining, they were assessed by a pathologist (single blind) using a light microscope (Zeiss, Germany) with magnification ×400 for evaluating average number of IgG^+^ cells in the region with maximum staining in the related slide. The results were reported with the following pattern: if the percent of positive cells was zero, the average would be reported as negative or none; if it was between 1 and 5, the average would be reported as A or slight; if it was between 6 and 10, the average would be reported as B or low; if it was between 11 and 25, the average would be reported as C or moderate; if it was between 26 and 50, the average would be reported as D; and finally, if the percent of positive cells was more than 50, the average would be reported as E or intense. To evaluate the distribution of these cells in different regions of tissue, each lesion was divided into three locations of inside and around epithelium, interjunction of epithelium-connective tissue, and within inflammatory cells. The study data then were analyzed by SPSS software version 13, using Mann-Whitney U test for comparison of the averages and regional distribution of IgG^+^ cells in the two groups. As our samples were only from prepared slides of patients, no ethical consideration was necessary.

## Results

**Figure 1:**
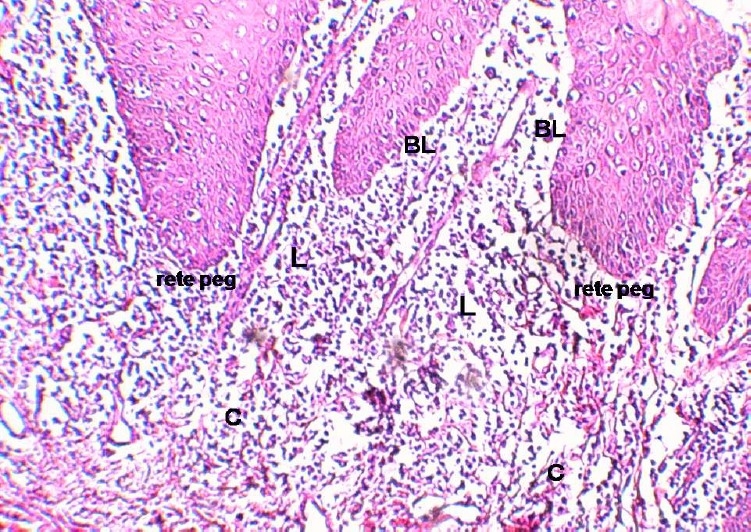
Oral lichen planus; BL: Basal layer, C: Connetive tissue, L: lymphocyte (H & E stain magnification X100).

**Figure 2:**
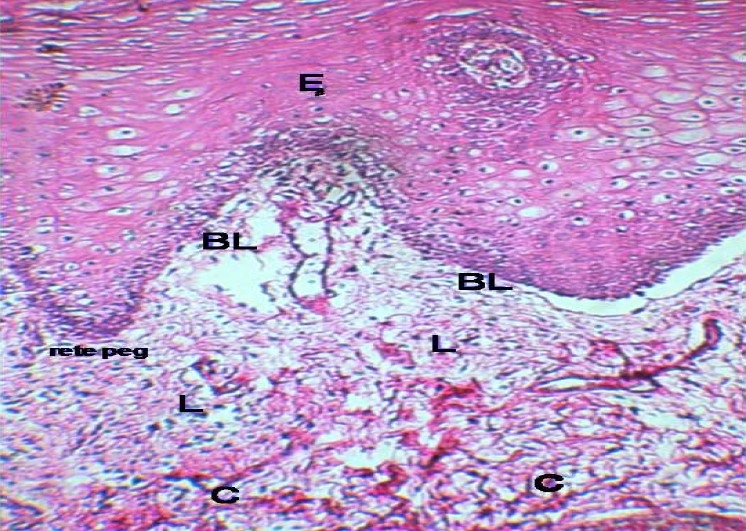
Oral lichenoid lesions; BL: Basal layer, C: Connetive tissue, E: Epithelium, L: lymphocyte (H & E stain magnification X100).

In this study, three regions from different locations were evaluated including “inside and around epithelium”, “interjunction of epithelium and connective tissue”, and “within epithelial cells” (figures 1 and 2). Then, the regional distribution of these cells was compared between the two groups and registered in a datasheet. The number of cells was ranked as 0-5%, 5-10%, 10-25%, 25-50% and more than 50% IgG^+^ cells in each region.

In OLP and also OLL, IgG^+^ cells were often found within epithelial cells. The sum of immuno-histochemical findings are summarized in table 1. At first, the distribution of IgG^+^ cells was evaluated in three regions of OLP and then, in OLL.

**Table 1:** Number of IgG^+^ cells distribution in O.L.P and O.L.L in different regions.

%(Number)	Lesion	Intra and around epithelium	Interjunction of epithelium-connective tissue	Within inflammatory cells
Oral Lichen Planus (N=30)	50<	0	0	6.7
	25-50	3.3	10.0	23.3
	10-25	6.7	26.7	50
	5-10	46.7	36.7	20.0
	0-5	43.3	26.7	0

Oral Lichenoid Lesions (N=30)	50<	0	0	3.3
	25-50	0	0	23.3
	10-25	10.0	26.7	43.3
	5-10	36.7	36.7	23.3
	0-5	53.3	36.7	6.7

There was a significant difference in distribution of IgG^+^ cells between the two regions of “inside and around epithelium” and “interjunction of epithelium and connective tissue” in OLP lesions (P = 0.034); the number of IgG^+^ cells was more in interjunction of epithelium and connective tissue. There was no significant difference in distribution of IgG^+^ cells between the two regions of “inside and around epithelium” and “interjunction of epithelium and connective tissue” in OLL (P = 0.10); the number of IgG^+^ cells between these regions was approximately similar. On the other hand, the difference of distributional region of IgG^+^ cells between the two regions of “inside and around epithelium” and “within inflammatory cells” in OLP was statistically significant (P = 0.00); i.e. the number of IgG^+^ cells in inflammatory cells was more than that in inside and around epithelium. Similarly, the difference of distributional region of IgG^+^ cells between the two regions of “inside and around epithelium” and “within inflammatory cells” in OLL was also statistically significant (P = 0.00); i.e. the number of IgG in inflammatory cells was more than that in inside and around epithelium.

The comparison of regional distribution of IgG^+^ cells between the two regions of “interjunction of epithelium and connective tissue” and “within inflammatory cells” in OLP showed that the differences were significant (P = 0.00); the number of IgG within inflammatory cells was more than that in interjunction of epithelium and connective tissue.

Finally, the comparison of regional distribution of IgG^+^ cells between the two regions of “interjunction of epithelium and connective tissue” and “within inflammatory cells” in OLL also showed that the differences were significant (P = 0.00); the number of IgG^+^cells within inflammatory cells was more that in interjunction of epithelium and connective tissue.

In the next part, the comparison of distribution of IgG^+^cells in each region was done between OLP and OLL. There was no significant difference in distribution of IgG^+^ cells in “interjunction of epithelium and connective tissue” between OLP and OLL (P = 0.49).

Similarly, the difference of distribution of IgG^+^ cells in “inside and around epithelium” region was not statistically significant (P = 0.25). Finally, the comparison of distribution of IgG^+^ cells “within inflammatory cells” between OLP and OLL didn’t show any significant difference (P = 0.40).

## Discussion

OLP and OLL are common diseases, which despite clinical and histopathological similarity, have different pathogenesis and prognosis. Lichen planus is a chronic systemic disease, which in some types (like erosive one) may be premalignant,^1^ but lichenoid lesion is an irritational disease which heals simply by removing irritational factors.^5^ Given the importance of differentiating these two lesions due to their completely different clinical process and prognosis,^2^ in this study the number and distribution of IgG^+^cells were taken as a base for comparison between the two lesions.

Results indicated significant differences between distribution of IgG^+^ cells in different regions of OLP and also of OLL, separately; i.e. “within inflammatory cells” of both lesions, IgG precipitation was more than that in the other two regions but the difference of distribution of this immunoglobulin between the two groups of OLP and OLL in none of these three regions was statistically significant.

The results of this study that verified the IgG precipitation in different regions of OLP and OLL by immunohistochemical staining, confirmed the study of Shousha et al.^7^ This was confirmed later by the study of Raychaudhuri et al.^14^ Furthermore, IgG precipitation in dermal lesions was indicated in the studies of Bouloc et al^11^ and Seishima et al.^12^ Flageul et al^15^ and Yoon et al^16^ demonstrated the IgG precipitation in dermal lichen planus and OLL by indirect immunofluorescence method, which is compatible with the results of our study; but, in none of the last studies, there was a comparison of immunoglobulin precipitation between the two lesions. That’s why the present study is unique.

The results indicated that the number and distribution of IgG^+^ cells cannot help differentiating OLP and OLL. We recommend a longer study with larger sample size. The current study was an immunofluorescence study to evaluate more precisely the differences of IgG distribution in different regions. Also, we suggest another study with serologic approach to assess IgE, IgM and IgG in blood of patients with OLP and OLL to be able to find a comprehensive method to compare and differentiate these two lesions.
